# Editorial: MSC Signaling in Regenerative Medicine

**DOI:** 10.3389/fbioe.2020.614561

**Published:** 2020-11-06

**Authors:** Martin J. Stoddart, Sandra Hofmann, Wolfgang Holnthoner

**Affiliations:** ^1^Regenerative Orthopaedics Program, AO Research Institute Davos, Davos, Switzerland; ^2^Department of Biomedical Engineering, Institute for Complex Molecular Systems, Eindhoven University of Technology, Eindhoven, Netherlands; ^3^Allgemeine Unfallversicherungsanstalt Research Centre, Ludwig Boltzmann Institute for Experimental and Clinical Traumatology, Vienna, Austria

**Keywords:** exosomes, microvesicles, secretome, mesenchymal stem cells, signaling

Originally designated mesenchymal stem cells (MSCs), decades of research have expanded the number of functions MSCs possess, in turn leading to various proposals as to what “MSC” should signify. While the earlier research focused on MSC ability to differentiate into various cell types, more recently their ability to control other cells by way of secreted molecules has been the subject of intense focus. This special issue brought together a collection of articles exploring the signaling aspects of MSCs. The review article from Marolt Presen et al. highlighted the wide functional range of MSCs, from cell transplantation to secretome, and their potential therapeutic use for bone regeneration. To enable clinical use, the ability to characterize the cells effectively will be crucial. Understanding the metabolic changes that underlie the MSC phenotype offers an opportunity to better monitor expansion and differentiation, as discussed in the review article from Sigmarsdóttir et al.. Pasztorek et al. investigated MSC phenotype after stimulating amniotic derived cells with platelet lysate instead of FBS. A detailed analysis of stress fiber formation and mitochondrial distribution demonstrated differences between the human platelet lysate and FBS. Similarly, changes in human infrapatellar fat pad-derived stem cell (IPFSC) phenotype were observed when the cells were cultured in the absence of fibronectin. CRISPR/Cas9 knockout cells, or IPFSCs grown on ECM deposited by knockout cells, displayed increased cell growth, but decreased differentiation.

The recent advent of extracellular vesicles (EV) led to a rise in publications focusing on the biology, characterization, isolation, and therapeutic possibilities. In the review article from Martin-Rufino et al., the ability of MSC-derived EV's to regulate the immune system was highlighted and potential mechanisms were discussed. Casado-Díaz et al. report in their review article on EVs derived from MSCs and their therapeutic potential for skin regeneration. They point out the feasibility of MSC-EVs, especially as a cell-free alternative aiming at skin wound healing. In the study of Mendes-Pinheiro et al., the secretome of bone marrow MSCs have been found to have neuroprotective effects in a rat model of Parkinson's disease and the authors suggest this is a feasible alternative to stem cell transplantation therapies. The study of Kastner et al. provided *in vitro* evidence that the MSC-derived secretome has the potential to aid the survival and regeneration of human cardiomyocytes that have been stressed under hypoxic conditions. Marinaro et al. report on the biological properties of EVs derived from endometrial MSCs, which are involved in regeneration and repair of the endometrium. They characterized the proteome and the microRNAome and found candidates involved in the immune regulation, apoptosis, and different signaling pathways, among others. With regards to angiogenesis, the study by Almeria et al. points out that EVs derived from MSCs cultured under hypoxic conditions appear to be functionally more potent to induce vascular tube formation than EVs derived from MSCs under normoxic conditions or the soluble factors in the media.

In another study by Nossin et al., the aim was to do the opposite, namely to inhibit blood vessel invasion in regenerating cartilage in an attempt to stabilize tissue engineered cartilage constructs for tissue repair and replacement. They investigated how chondrogenically differentiated BMSCs could be prevented from maturing into bone through the endochondral pathway by investigating the pro-angiogenic potential of the secretome. Their results suggest that the endochondral ossification process could be prevented by inhibiting specific pro-angiogenic factors such as Indian Hedgehog and Serpin E1. The effect of soluble mediators can also be seen during the MSC expansion phase. Voskamp et al. demonstrated an increased chondrogenic potential of MSCs that were exposed to TNFα during cell expansion, even when the TNFα was still present during the differentiation phase. This offers a potential mechanism to improve chondrogenesis in inflamed sites.

Palomares Cabeza et al. studied the potential of MSCs derived from pediatric patients over MSCs from adults to modulate the immune response. They successfully showed that pediatric MSCs show immunomodulatory effects on allogeneic T- and B-cells and that those properties can be potentiated by inflammatory stimuli. All in all, this could have important implications in implant engraftment and the avoidance of a strong immune reaction.

## Author Contributions

All authors listed have made a substantial, direct and intellectual contribution to the work, and approved it for publication.

## Conflict of Interest

The authors declare that the research was conducted in the absence of any commercial or financial relationships that could be construed as a potential conflict of interest.

